# Primary cardiac angiosarcoma: a prolonged response to surgical resection followed by concurrent chemoradiotherapy with docetaxel

**DOI:** 10.1186/s40064-016-2248-8

**Published:** 2016-05-17

**Authors:** Youngwoo Jang, Joonhwan Kim, Jung Woo Shim, Eun Young Kim, Jungkeun Moon, Jungsuk An, Yang Bin Jeon, Kyu Chan Lee, Hee Kyung Ahn

**Affiliations:** Department of Internal Medicine, Gachon University Gil Medical Center, 1198 Guwol-dong, Namdong-gu, Incheon, 405-760 Republic of Korea; Department of Radiology, Gachon University Gil Medical Center, Incheon, Republic of Korea; Department of Pathology, Gachon University Gil Medical Center, Incheon, Republic of Korea; Department of Thoracic and Cardiovascular Surgery, Gachon University Gil Medical Center, Incheon, Republic of Korea; Department of Radiation Oncology, Gachon University Gil Medical Center, Incheon, Republic of Korea

**Keywords:** Primary cardiac angiosarcoma, Chemotherapy, Docetaxel, Radiotherapy, Surgery

## Abstract

**Introduction:**

Primary cardiac cancer is a very rare disease, among which primary cardiac angiosarcoma is one of the most frequent type and is characterized by extremely poor prognosis without established optimal treatment.

**Case description:**

Here we report a case of primary cardiac angiosarcoma with hemorrhagic pericardial effusion who achieved a durable response with tumor excision followed by concurrent chemoradiotherapy with docetaxel. A sixty year old man was presented with dyspnea and was diagnosed with primary cardiac angiosarcoma with hemorrhagic pericardial effusion. After surgical excision of primary tumor with microscopic residual disease followed by concurrent chemoradiotherapy with docetaxel, the patient showed durable response of progression free survival of 12 months.

**Discussion and evaluation:**

This case shows benefit of concurrent chemoradiotherapy with taxane. Further investigation of aggressive multimodal treatment strategy is warranted for primary cardiac angiosarcoma with pauci-metastasis even when achievement of complete resection seems unlikely.

## Background

 Primary cardiac tumors are rare with frequency of 0.001–0.030 %, of which a quarter is malignant and 95 % of malignant cardiac tumors are sarcomas (Butany et al. 2005). Primary cardiac angiosarcoma is the most common type of cardiac sarcomas and has the worst prognosis with median survival less than 1 year (Isambert et al. 2014; Llombart-Cussac et al. 1998; Simpson et al. 2008; Kim et al. 2008). Standard treatment for primary cardiac angiosarcoma is not clear. Here we report a case of primary cardiac angiosarcoma with hemorrhagic pericardial effusion who achieved a durable response with multidisciplinary approach.

## Case report

**Fig. 1:**
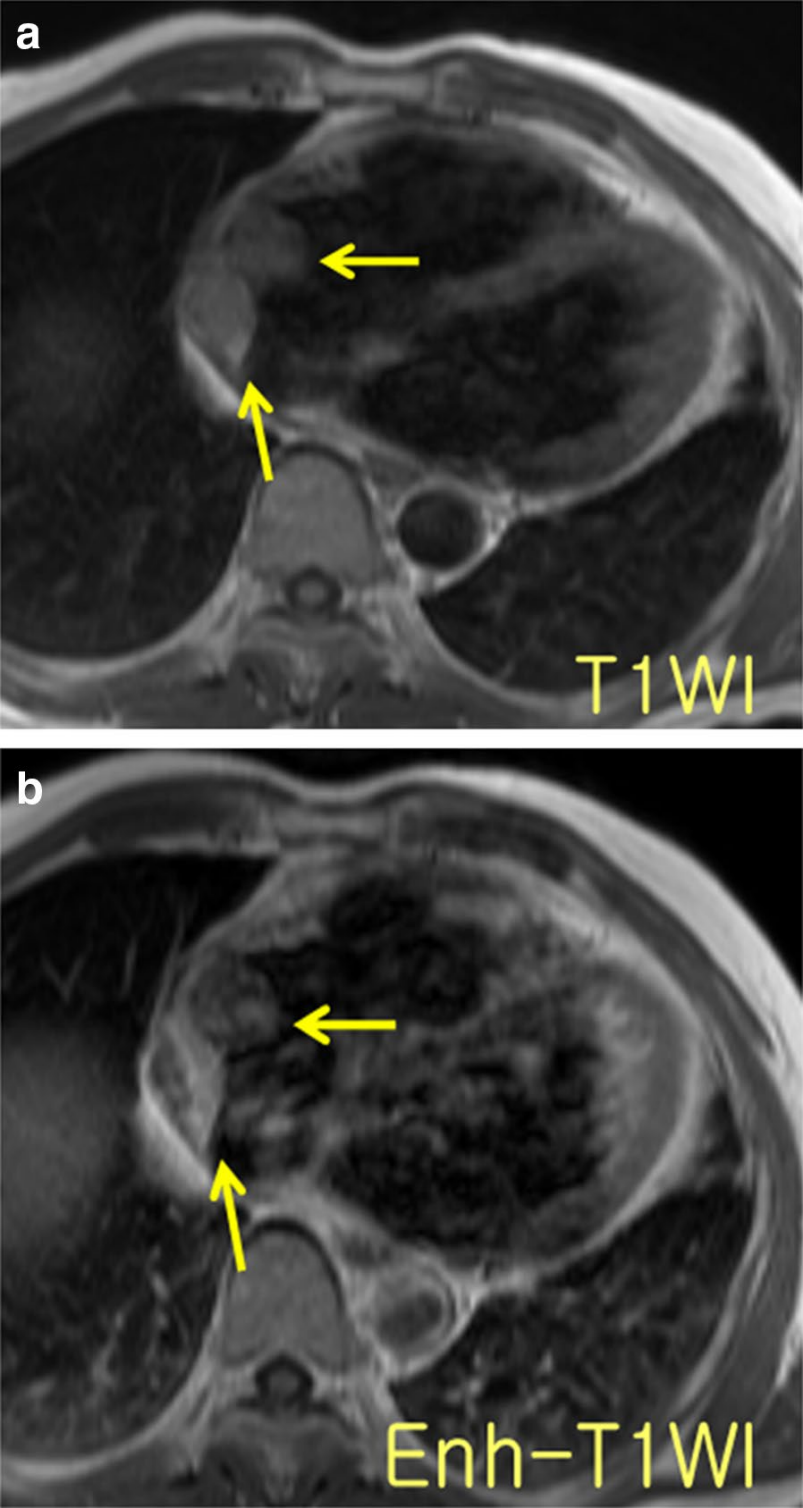
**a** Double inversion-recovery T1-weighted image demonstrates a relatively isointense lobulated mass in the free wall of right atrium. **b** Gadolinium-enhanced T1-weighted image shows heterogeneous enhancement of the mass. Within the mass, some portions had no enhancement, representing intra-tumoral thrombosis or necrosis

A sixty-year-old man presented to an emergency room with rapidly progressing shortness of breath and chest discomfort in April 2013. Echocardiography revealed cardiac tamponade with a large amount of pericardial effusion and a mass in right atrium. Four hundred and twenty mL of bloody fluid was drained by pericardiocentesis. Laboratory analysis of the pericardial effusion failed because of the high viscosity. The patient was referred to our institution. Cardiac magnetic resonance imaging (MRI) revealed a 4.5 × 3.5 cm sized infiltrative mass in the right atrium (Fig. 1). In 18F-Fludeoxyglucose-positron emission tomography (FDG-PET) scan, the cardiac mass showed high FDG-uptake without any evidence of distant metastasis.

Surgical resection of cardiac tumor was performed, in which the pericardium was thickened without adhesion, and a blackish friable protruding mass was observed within the right atrium near the inferior vena cava. The pathology diagnosis was angiosarcoma. There was no gross residual disease on surgical field and on postoperative cardiac MRI, although microscopic examination showed tumor involvement of the resection margin.

The patient was treated with adjuvant concurrent chemoradiotherapy (CRT) 5000 cGys/30 fractions with five cycles of weekly docetaxel (25 mg/m^2^). He tolerated the treatment very well except for several episodes of palpitation, which started after surgery and before initiation of CRT. Paroxysmal atrial fibrillation was diagnosed that subsided after completion of CRT. There was no evidence of recurrence until April 2014, when three liver metastases were found on liver MRI. The patient was treated with hepatic metastasectomy and palliative chemotherapy with weekly paclitaxel for 16 weeks until when new liver metastases were noted in January 2015. Then he subsequently received pazopanib for another 6 months. He eventually died of disease progression in October 2015; overall survival was 32 months.

## Discussion and conclusion

Optimal treatment strategy for primary cardiac sarcomas is not established. Complete surgical resection, while challenging, is associated with better prognosis (Isambert et al. 2014; Llombart-Cussac et al. 1998; Simpson et al. 2008). A few case reports showed successful treatment of this aggressive disease with multidisciplinary treatment (Baay et al. 1994; Kakizaki et al. 1997), and afterwards many case series suggested a favorable role of multimodal therapy for improved survival (Isambert et al. 2014; Simpson et al. 2008; Randhawa et al. 2014; Barreiro et al. 2013; Bakaeen et al. 2009). A recent series of primary cardiac sarcomas describes a role of surgical resection in pauci-metastatic patients and radiotherapy in cases with incomplete resection or no surgery for prolonging survival (Isambert et al. 2014).

Adjuvant chemotherapy with doxorubicin containing regimen did not seem to have benefit in patients with primary cardiac sarcomas including six angiosarcomas (Llombart-Cussac et al. 1998). Weekly paclitaxel showed its efficacy in unresectable angiosarcoma with overall response rate of 18 % and clinical benefit rate of 74 % in a phase 2 trial (Penel et al. 2008), although it is not clear whether primary cardiac angiosarcomas were included in the enrolled eight visceral primary angiosarcomas. Recent case reports have shown the possible benefit of taxane in primary cardiac angiosarcomas. Concurrent CRT with taxane containing regimen [paclitaxel and carboplatin (Hata et al. 2011), paclitaxel (Fehr et al. 2010), and docetaxel (Nakamura-Horigome et al. 2008; Suderman et al. 2011)] has been attempted in several inoperable cases with clinical benefit. The benefit of surgical resection followed by sequential taxane containing chemotherapy (gemcitabine and docetaxel) and radiotherapy (Bellitti et al. 2013), or palliative paclitaxel (Ong et al. 2012) was reported. In line with previous reports, although visible tumor response was not assessed in our case because CRT with docetaxel was administered as adjuvant treatment, the patient achieved durable progression-free survival of 1 year despite the presence of hemorrhagic pericardial effusion at diagnosis and R1 resection. Tolerability of the CRT was good without any grade 3 or 4 adverse events. Moreover, prolonged survival after the recurrence was observed with weekly paclitaxel and liver metastasectomy.

This case warrants further investigation of CRT with docetaxel and aggressive multimodal treatment strategy for primary cardiac angiosarcoma with pauci-metastasis even when achievement of complete resection seems unlikely.
